# How do specialist surgeons treat the atrophic tooth gap? A vignette-based study among maxillofacial and oral surgeons

**DOI:** 10.1186/s12903-021-01688-9

**Published:** 2021-07-03

**Authors:** Michael Korsch, Winfried Walther, Bernt-Peter Robra, Aynur Sahin, Matthias Hannig, Andreas Bartols

**Affiliations:** 1 Dental Academy for Continuing Professional Development, Lorenzstrasse 7, 76135 Karlsruhe, Germany; 2 Clinic of Operative Dentistry, Periodontology and Preventive Dentistry, University Hospital, Saarland University, Building 73, 66421 Homburg, Germany; 3 Center for Implantology and Oral Surgery, 69120 Heidelberg, Germany; 4 Institute of Social Medicine and Health Services Research, Otto-von-Guericke-University of Magdeburg, Magdeburg, Germany; 5 Blumenstrasse 5, 69115 Heidelberg, Germany; 6 Clinic for Conservative Dentistry and Periodontology, School for Dental Medicine, Christian-Albrechts-University Kiel, Kiel, Germany

**Keywords:** Dental implant, Specialists, Oral surgeon, Maxillofacial surgeon, Pre-implantological treatment, Bone augmentation, Tooth gap

## Abstract

**Background:**

There is little information available regarding the decision-making process of clinicians, especially in the choice of therapy for a severely atrophic tooth gap. The aim of this research was to use case vignettes to determine the influence of possible factors on the decision making of maxillofacial and oral surgeons.

**Methods:**

A total of 250 maxillofacial (MFS) and oral (OS) surgeons in southern Germany were surveyed for atrophic single- or multiple-tooth gap with the help of case vignettes. The influence of different determinants on the therapy decision was investigated. Two case vignettes were designed for this purpose: vignette 1 with determinants “patient age” and “endocarditis prophylaxis” and vignette 2 with determinants “anxiety” and “bisphosphonate therapy”. Furthermore, the specialist designation was assessed for both. The options available to achieve a sufficient implant site were "bone split", "bone block", "augmentation with bone substitute material" and "bone resection". Therapy was either recommended or rejected based on principle.

**Results:**

A total of 117 participants returned the questionnaire: 68 (58%) were OS and 49 (42%) MFS. “Patient age” and “patient anxiety” were not significantly associated with any therapy decision. However, required “endocarditis prophylaxis” led to significantly higher refusal rates for "bone split", "bone block" and "bone replacement material" and to higher rates of general refusal of a therapy. “Bisphosphonate therapy” was significantly associated with general refusal of therapy, but with no significant correlation with different therapy options. In vignette 1, OS refused therapy significantly more often than MFS, though there was no association with the specialist designation for other therapy modalities. In vignette 2, specialty was not significantly associated with the therapy decision.

**Conclusion:**

“Patient age” as well as “patient anxiety” appear to have no or little influence on the treatment decision for severely atrophic single- or multiple-tooth gap by specialist surgeons. Surgeons more often refuse treatment for patients with endocarditis prophylaxis and bisphosphonate therapy.

**Supplementary Information:**

The online version contains supplementary material available at 10.1186/s12903-021-01688-9.

## Background

There are basically three pre-implantological methods employed to achieve a sufficiently wide implant site in a severely atrophied alveolar ridge: additive, expansive and subtractive methods. Although additive techniques achieve widening of the alveolar ridge by placing an augmentation material on top of it, expansive techniques do so by splitting the alveolar ridge into two parts and stretching them to the desired width. When using subtractive techniques, the narrow part of the jaw is resected until a sufficiently wide alveolar ridge is achieved at the base [1].

Commonly used additive techniques are augmentation with bone substitute material and bone block grafting [2–4]. Bone splitting is applied as an expansive techniques [5]. Simultaneous implantation is usually possible in bone resection; in contrast, one-stage surgery is hardly ever possible or only partially with other techniques of alveolar ridge widening. Consequently, the implant must be placed in a second operation, which extends the treatment time and increases the operative risk.

To date, the cases in which specialist surgeons favour specific techniques of alveolar ridge enlargement remain unclear. Moreover, there is little research regarding the influence on decision making of indications, underlying diseases and the patients' fear of surgery. To analyse the influence of different clinical parameters on therapy decisions, the scientific instrument of case vignettes is frequently employed in the area of health care research. These vignettes are typified case descriptions for the experimental investigation of care decisions [6, 7]. Case vignettes have comparable significance with regard to medical practice as a systematic evaluation of medical records or standardized patients [8]. Furthermore, vignette-based surveys are particularly suitable for the evaluation of complex health care problems and can be used specifically for quality management purposes [6]. Additionally, compared to other instruments, this approach has the advantage of experimentally controllable parameters. A vignette contains either an authentic individual case presentation (casuistic vignette) or a typified case presentation systematically compiled from medical and social factors (systematic case vignette) [6].

Through the use of case vignettes, this study aimed to analyse to what extent the factors patient "age", "co-morbidity" (endocarditis prophylaxis or bisphosphonate therapy) and "fear of surgery" influence the treatment decision for severely atrophic single- or multiple-tooth gap by two groups of specialists (maxillofacial vs. oral surgeon).

## Methods

Implantological and augmentative procedures may be performed by any dentist, though advanced training in implantology is recommended to acquire specialist knowledge. Maxillofacial surgeons as well as oral surgeons receive a well-founded education in the field of implantology within the scope of their training as a specialist surgeon.

For this study, a questionnaire in paper form was prepared and sent to 250 maxillofacial and oral surgeons, exclusively involving resident surgeons in private practice (oral and maxillofacial surgeons) with the authorization to conduct specialist training in oral surgery. The eligibility to train oral surgeons ensured that the respondents were experienced practitioners. In all cases, telephone contact was established before the questionnaire was sent. In the telephone conversation, the questionnaire was explained, and information about the study objective was provided; willingness to participate in the present study was also requested. If contact was not possible even after several attempts, the interviewee received the questionnaire without advance notice. The study was reviewed and approved by the Ethics Committee of the Medical Association of the Saarland (Ref. No.: 133/11).

### Structure of the questionnaire

The first part of the questionnaire involved physician and practice-related characteristics. The part of the questionnaire relevant for this study consisted of clinical case vignettes. For each case vignette, the study participant was asked to choose his/her preferred therapy. The original questionnaire is available as supplementary information (Additional file 1: S1 English and Additional file 2: S1 Original).

The present publication addresses clinical case vignettes for the treatment of severely atrophic single- or multiple-tooth gaps**.**

### Clinical case vignettes

To survey the indication practices of the interviewees in implantological therapy, the case vignettes consisted of two real cases of gap switching with a narrow alveolar ridge. The case vignettes included medical anamnesis, clinical findings and X-rays (orthopantomograms and cone beam computed tomography (CBCT) excerpts). The X-rays were used for visualization and were selected such that they could be easily judged when printed on paper. The two cases corresponded to a frequent clinical indication in routine implant therapy.

### Variable descriptors

Both of the two case vignettes had two variable descriptors in the medical anamnesis. The first case vignette dealt with "patient age" and the co-morbidity "endocarditis prophylaxis"; the second addressed the co-morbidity "bisphosphonate therapy" and "surgery anxiety" of the patient. In each of the vignettes, the descriptors had one of two characteristics. Thus, a low or high patient age, endocarditis prophylaxis necessary or not, bisphosphonate therapy present or not and fear of surgery present or not were given; four combinations were possible for each vignette. A random generator was used to achieve an independent distribution of all vignette characteristics in the vignette sets, and this planned variance of individual determinants enables the identification of decision patterns for the preference of a particular therapy. This was intended to reveal whether the therapy decision is different in younger and older patients, in the case of necessary endocarditis prophylaxis or in the presence of bisphosphonate therapy and patients without co-morbidity and in anxious patients and those without fear of surgery. In addition, the influence of the specialist designation on the decision was assessed. The previously mentioned techniques for therapy of the narrow alveolar ridge, bone split, bone block, augmentation with bone substitute material and bone resection were given as potential choices. These techniques covered the three methods for achieving a wide implant site. A therapy could be generally approved or rejected.

To assess the case vignettes for practicability (comprehensibility, consistency of content of the findings and measures), they were tested beforehand by a total of five surgeons for comprehensibility and clinical relevance.

### Description of the clinical case vignettes

#### Case vignette 1 ("single-tooth gap")

This clinical case vignette established the influence of age and need for endocarditis prophylaxis on clinical decision making.

**Fig. 1:**
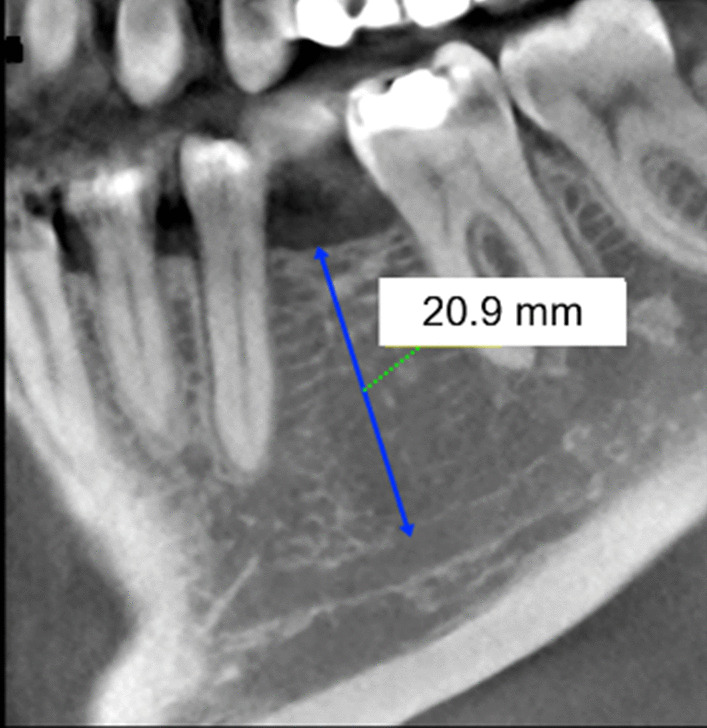
Sagittal plane from the CBCT of region 36 for case vignette 1. The alveolar ridge height is sufficient to place an implant

**Fig. 2:**
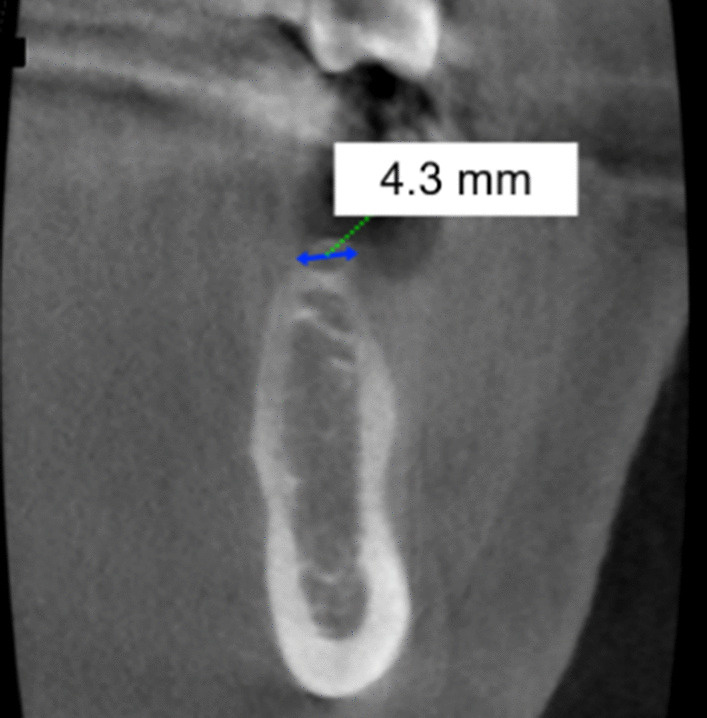
Cross sections from the CBCT of region 36 for case vignette 1. The alveolar ridge is atrophied in the crestal region

It concerned a patient in whom tooth 36 had been missing for one year. The patient was a non-smoker and was very critical of the procedure. The X-ray findings [orthopantomogram and CBCT, (Figs. 1, 2)] showed an atrophic alveolar region 36 with sufficient bone height. The referring physician's wish was a single-tooth restoration with an implant to replace tooth 36.

The following variants were built into the vignettes:

- Combination 1: *Age of the patient 52 years, no systemic diseases*
- Combination 2: *Age of the patient 52 years, endocarditis prophylaxis required because of an artificial heart valve*
- Combination 3: *Age of the patient 76 years, no systemic diseases*
- Combination 4: *Age of the patient 76 years, endocarditis prophylaxis required because of an artificial heart valve*

#### Case vignette 2 ("multiple-teeth gap", three teeth missing)

**Fig. 3:**
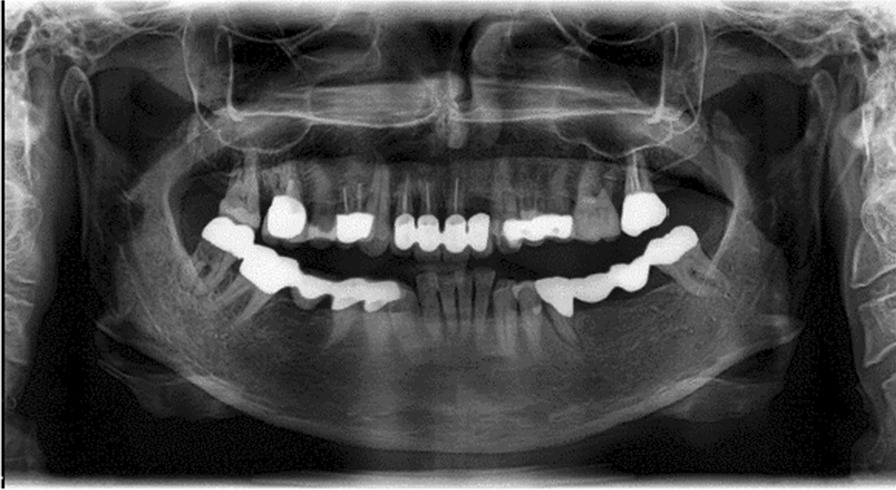
Panoramic tomography for case vignette 2 before extraction of tooth 35. The bone height in region 35–37 is sufficient for placing implants

**Fig. 4:**
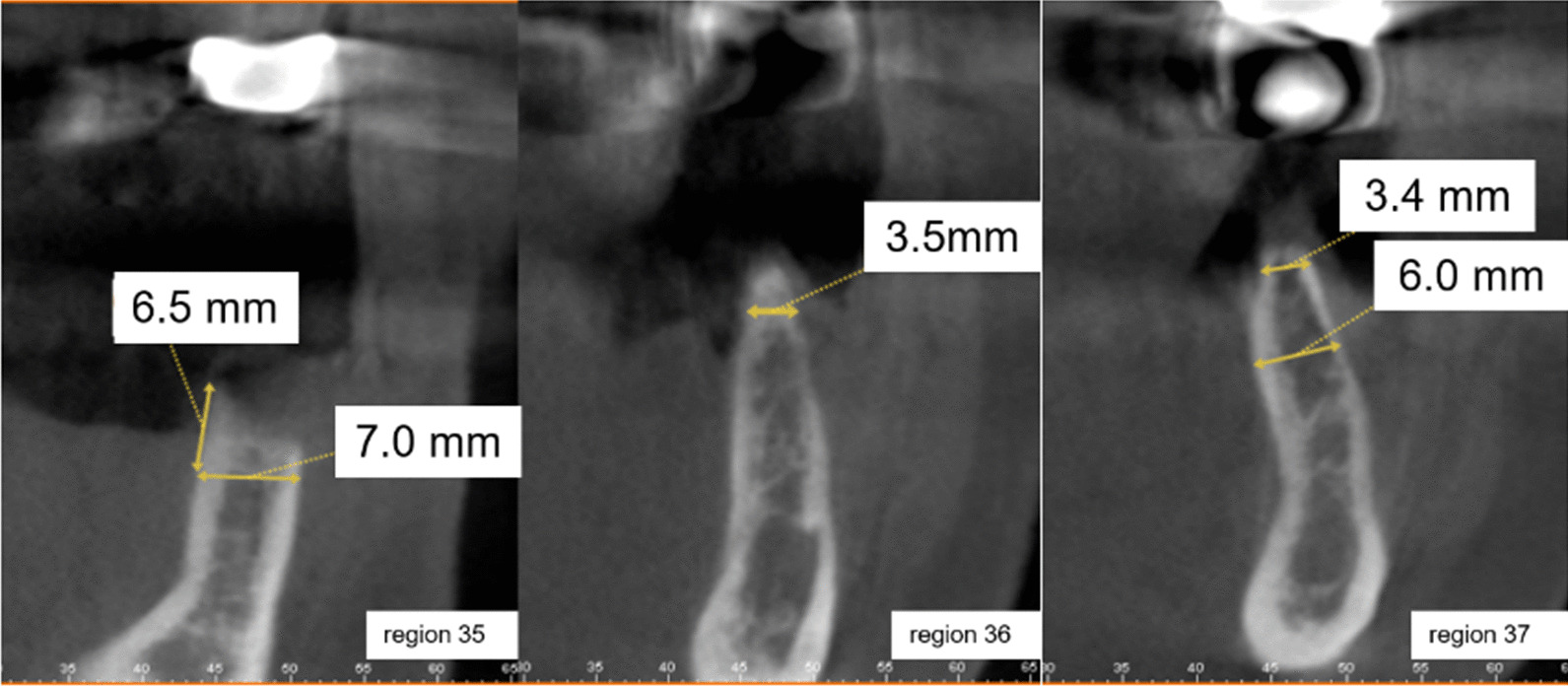
Cross-sections of regions 35–37 from the CBCT at 5 months after extraction of tooth 35. The alveolar ridge in region 35–37 is narrow in the crestal area and insufficient for implantation without pre-implantological treatment

This clinical case vignette examined the influence of the presence of bisphosphonate therapy (Fosamax) and the patient's attitude towards the intended treatment. The female patient was 57 years old. Tooth 35 could not be preserved due to a longitudinal fracture and had to be removed. The patient could not cope with the provisional prosthesis in region 35–37. Radiographs (orthopantomogram and CBCT, Figs. 3, 4) showed sufficient bone height in region 35–37. The alveolar ridge was atrophied. The referring dentist proposed fixed restoration in the lower jaw.

The following variants were built into the vignettes:

- Combination 1: *No systemic diseases, the patient was positive about the procedure*
- Combination 2: *No systemic diseases, the patient was very anxious*
- Combination 3: *Fosamax medication, the patient was positive about the procedure*
- Combination 4: *Fosamax medication, the patient was very anxious*

For both vignettes, the study participants were asked to choose their preferred surgical procedure. For this purpose, the five aforementioned response options were given: bone split, bone block, augmentation with bone substitute material, bone resection and no therapy.

It was possible to select "yes", "not at all", or "possibly" for the respective therapy options. In this case, "yes" denoted "this option represents the therapy of my choice", "by no means" denoted "this therapy is ruled out", and "possibly" denoted "I am considering this option; I will decide intraoperatively". In addition, the respondents had the opportunity to write down free answers in the section "other".

### Data evaluation

The data from the questionnaires were collected using Microsoft Excel and analysed using IBM SPSS Statistics 21 (IBM SPSS Statistics, IBM, Armonk, New York, United States) in Windows XP. Microsoft PowerPoint was used to create graphs.

The analysis was performed with complete data sets. Missing information from the participants was excluded on a case-by-case basis. Logistic regressions for binary dependent variables were applied. Assessment of whether the respondent's specialty is related to the preferred care was performed. A probability of error of *p* < .05 was interpreted as significant. The raw data are available as a supplementary file (Additional file 3: S2).

## Results

A total of 117 of the 250 questionnaires sent were returned, with a response rate of 46.8%. The combinations of characteristics of the responses to the 117 questionnaires were distributed similarly to the sample sent (approximately a quarter each) (Table 1).

**Table 1:** Distribution of the vignette combinations of the returned questionnaires (N = 117)

	Vignette 1	Vignette 2
Combination 1	31 (26.5%)	33 (28.2%)
Combination 2	37 (31.6%)	35 (29.9%)
Combination 3	22 (18.8%)	26 (22.2%)
Combination 4	27 (23.1%)	23 (19.7%)
Chi-Square test	n.s.	n.s.

### Outcomes for case vignette 1 (single-tooth gap)

**Fig. 5:**
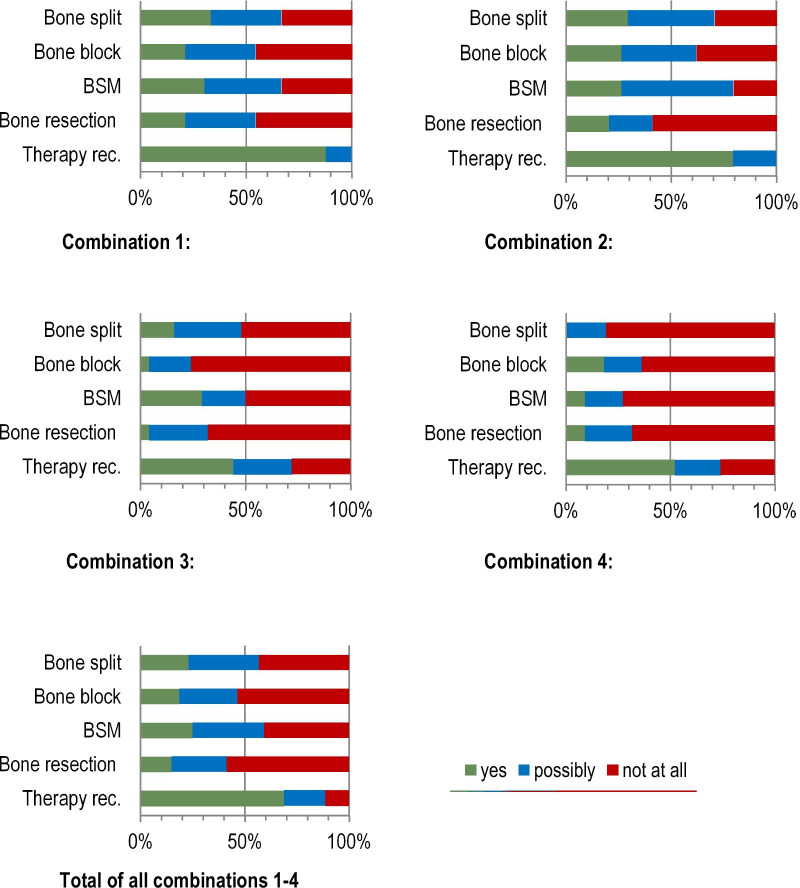
Case vignette 1: Preferred therapy decisions of the surgeons surveyed for the restoration of the atrophied single-tooth gap according to the four different combinations of "patient age" and "endocarditis prophylaxis". **Combination 1:** Age of the patient 52 years, no systemic diseases. **Combination 2:** Age of the patient 52 years, endocarditis prophylaxis required because of an artificial heart valve. **Combination 3:** Age of the patient 76 years, no systemic diseases. **Combination 4:** Age of the patient 76 years, endocarditis prophylaxis required because of an artificial heart valve

The analysis showed (Fig. 5) that 70% of the participants were in favour of therapy. Only 13.6% rejected any treatment, and 16.4% would "possibly" treat. 40.4% of the respondents indicated bone substitute material as the therapy of choice. However, 29.4% rejected bone graft substitutes. In addition, bone split was rejected by 52.4%, and rejection for bone block (68.0%) and bone resection (66.7%) was even higher.

The binary logistic regression showed a significant association of the response option "therapy recommended" with the specialist designation (maxillofacial surgeon vs. oral surgeon), here parameterized as a dependent variable (Table 2). Specifically, oral surgeons more frequently rejected therapy than maxillofacial surgeons (17.3% vs. 8.3%). no association with the specialist designation.

**Table 2:** Association between the dependent variable “specialist designation” (maxillofacial vs. oral surgeon) and the independent variables “bone split”, “bone block”, “bone substitute material”, “bone resection” and “therapy recommended” for case vignette 1

	Regression coefficient B	Standard error	Forest	df	Sig.	Exp(B)
Bone split	− .686	.588	1.359	1	.244	.504
Bone block	− .287	.737	.152	1	.697	.751
Bone substitute material	− .666	.468	2.026	1	.155	.514
Bone resection	− .461	.835	.305	1	.581	.631
Therapy recommended	− 1.559	.764	4.166	1	**.041**	.210
Constant	.355	.388	.840	1	.359	1.427

Bold values indicate statistically significant differences (*p* < 0.05)

Nagelkerkes R-squared .50

The evaluation also showed no significant association between possible therapy options and patient age (Table 3).

**Table 3:** Association between the dependent variable “patient age” (52 years vs. 76 years) and the independent variables “bone split”, “bone block”, “bone substitute material”, “bone resection” and “therapy recommended” for case vignette 1

	Regression coefficient B	Standard error	Forest	df	Sig.	Exp(B)
Bone split	− .452	.567	.636	1	.425	.636
Bone block	.214	.767	.078	1	.781	1.238
Bone substitute material	.268	.481	.311	1	.577	1.307
Bone resection	1.273	1.129	1.270	1	.260	3.572
Therapy recommended	− .917	.692	1.755	1	.185	.400
Constant	.447	.393	1.296	1	.255	1.564

Nagelkerkes R-squared .50

However, a required endocarditis prophylaxis was significantly associated with the therapy options "bone split", "bone block", "bone replacement material" and with the general rejection of treatment (Table 4). In general, the therapy options "bone split", "bone block" and "bone replacement material" were less frequently favoured in the case of necessary endocarditis prophylaxis and rejection of treatment was more frequent (see also Fig. 5). There was no association with the therapy option "bone resection" (Table 4).

**Table 4:** Association between the dependent variable “endocarditis prophylaxis” (necessary vs. not necessary) and the independent variables “bone split”, “bone block”, “bone substitute material”, “bone resection” and “therapy recommended” for case vignette 1

	Regression coefficient B	Standard error	Forest	df	Sig.	Exp(B)
Bone split	1.588	.658	5.834	1	**.016**	4.896
Bone block	1.738	.844	4.240	1	**.039**	5.685
Bone substitute material	1.610	.545	8.735	1	**.003**	5.005
Bone resection	.887	.903	.963	1	.326	2.427
Therapy recommended	1.489	.744	4.010	1	**.045**	4.433
Constant	− 1.643	.493	11.092	1	.001	.193

Bold values indicate statistically significant differences (*p* < 0.05)

Nagelkerkes R-squared .50

Among "other", the most frequently mentioned options were bridge restoration (N = 14, 12%), diameter-reduced implants (N = 6, 5.1%) and vestibular placement of particulate bone (N = 5, 4.3%). Orthodontic gap opening (1.7%) was reported twice, and plasma rich growth factor (PRGF) (.9%), shell technique (.9%) and Astra Tech Profile implant (Dentsply Sirona, Mölndal, Sweden) (.9%) were each reported once as therapy options.

### Outcomes for case vignette 2 (multiple-teeth gap)

**Fig. 6:**
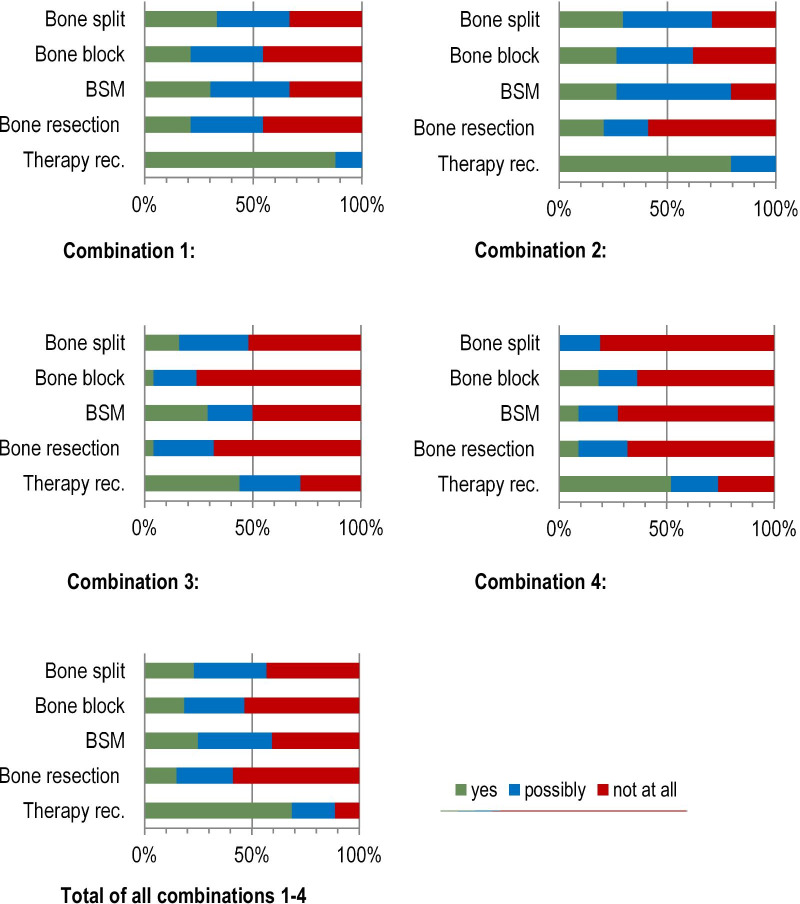
Case vignette 2: preferred therapy decisions of the surgeons surveyed for the restoration of the edentulous severely atrophied mandible according to the four different combinations of "surgery anxiety" and "bisphosphonate therapy". Combination 1: no systemic diseases, the patient was positive about the procedure. Combination 2: no systemic diseases, the patient was very anxious. Combination 3: Fosamax medication, the patient was positive about the procedure. Combination 4: Fosamax medication, the patient was very anxious. *BSM* bone substitute material, *Therapy rec.* therapy recommended

A total of 68.7% of the participants in the survey were in favour of therapy in case vignette 2, whereas only 11.3% would refuse treatment (Fig. 6); 20% of all surgeons would "possibly" provide therapy. The favoured treatment options were distributed relatively homogeneously among the possible choices (bone split 22.9%, bone block 18.4%, bone substitute material 24.8% and bone resection 14.9%), with bone resection (58.8%) and bone block (53.5%) being the most frequently rejected options.

The specialist designation (maxillofacial vs. oral surgeons) was not associated with the therapy choice according to binary logistic regression (Table 5). In addition, patient anxiety did not have a significant influence on the therapy decision (Table 6).

**Table 5:** Association between the dependent variable “specialist designation” (maxillofacial vs. oral surgeon) and the independent variables “bone split”, “bone block”, “bone substitute material”, “bone resection” and “therapy recommended” for case vignette 2

	Regression coefficient B	Standard error	Forest	df	Sig.	Exp(B)
Bone split	− .030	.235	.016	1	.898	.970
Bone block	.218	.237	.846	1	.358	1.244
Bone substitute material	.011	.249	.002	1	.964	1.011
Bone resection	.341	.230	2.200	1	.138	1.406
Therapy recommended	.078	.243	.104	1	.747	1.081
Constant	− .715	.380	3.538	1	.060	.489

Nagelkerkes R-squared .50

**Table 6:** Association between the dependent variable "surgery anxiety" (yes vs. no) and the independent variables “bone split”, “bone block”, “bone substitute material”, “bone resection” and “therapy recommended” for case vignette 2

	Regression coefficient B	Standard error	Forest	df	Sig.	Exp(B)
Bone split	.114	.234	.236	1	.627	1.120
Bone block	− .170	.236	.523	1	.470	.843
Bone substitute material	− .191	.248	.593	1	.441	.826
Bone resection	.257	.229	1.257	1	.262	1.293
Therapy recommended	− .150	.244	.376	1	.540	.861
Constant	.144	.368	.153	1	.696	1.155

Nagelkerkes R-squared .50

Bisphosphonate therapy showed no correlation with the different therapy options, but bisphosphonate therapy was significantly associated with the general refusal of treatment (Table 7). Although 44% (combination 3) and 52.2% (combination 4) of the surgeons were in favour of treatment with bisphosphonate therapy, the proportions were 87.9% (combination 1) and 79.4% (combination 2) in the absence of bisphosphonate therapy (Fig. 6).

**Table 7:** Association between the dependent variable "bisphosphonate therapy" (present vs. not present) and the independent variables “bone split”, “bone block”, “bone substitute material”, “bone resection” and “therapy recommended” for case vignette 2

	Regression coefficient B	Standard error	Forest	df	Sig.	Exp(B)
Bone split	.510	.266	3.679	1	.055	1.665
Bone block	.505	.276	3.352	1	.067	1.657
Bone substitute material	.510	.274	3.453	1	.063	1.665
Bone resection	.038	.265	.021	1	.885	1.039
Therapy recommended	− .735	.274	7.195	1	**.007**	.480
Constant	− .461	.400	1.330	1	.249	.630

Bold values indicate statistically significant differences (*p* < 0.05)

Nagelkerkes R-squared .50

Among "others", the most frequent response was the use of diameter-reduced implants (N = 4, 3.4%), conventional dentures (bridge and removable dentures) (N = 4, 3.4%), and vestibular placement of particulate bone (N = 3, 2.6%). A shell technique was mentioned twice (1.7%) as a therapy option and PRGF (.9%) and Astra Tech Profile implant (.9%) once each.

## Discussion

The present case vignette study was designed to identify which treatment options for single-tooth or multiple-tooth gap are preferred by specialists and the influence that a modification of descriptors in the case presentation has on this decision. It was thus shown that co-morbidities such as required endocarditis prophylaxis and bisphosphonate therapy tended to lead to the rejection of certain therapy techniques as well as to a general refusal of therapy. Interestingly, neither patient age nor fear of surgery influenced the surgeons' decision.

Written case presentations can be used to explore the decision-making process of physicians and their clinical competence [9]. Parameters such as age, (co)morbidity, patient attitude and smoking behaviour all influence the physician's choice of therapy [10]. In addition, the knowledge and usual routines of surgeons influence decision making [11].

The participants of this study were all maxillofacial and oral surgeons. To increase their motivation to participate in the survey, they were contacted by telephone, informed about the survey and asked for their consent, and the response rate was 46.8%. A study on postal surveys in Germany showed that those without incentives lead to a response rate of only 28% but that a reward had a considerable positive effect (52%) [12]. The response rate in the present study without incentives must therefore be regarded as high. A non-responder analysis was not possible in this study.

For case vignette 1, 70% of the respondents recommended therapy. "Bone replacement material" was chosen most frequently, followed by "bone split", with an obvious preference. "Bone resection" was chosen least frequently. Although bone resection creates a sufficient implant site using simple methods, the reduction in bone height means that tooth restoration in the gap has to be designed under unfavourable conditions. In fact, the crown length of the implant becomes longer than of the natural neighbouring teeth, and as a result, oral hygiene can be more difficult. This may be an important reason for the rare choice of this therapy for a single-tooth gap. Nevertheless, this form of therapy can be of great advantage for the edentulous jaw, as simultaneous implant placement is possible and the influence of the altered bone height on the prosthetic restoration is minimal [1].

Moreover, the specialist designation of the surgeon was only significantly associated with the general therapy decision: oral surgeons tended to avoid therapy more than maxillofacial surgeons. The reasons for this could not be determined based on the study data and therefore remain speculative. Additionally, patient age only played a minor role in the therapy decision. In general, the therapy options "bone split", "bone block" and "bone replacement material" were less frequently favoured in cases where endocarditis prophylaxis was required; the tendency to refuse therapy in endocarditis cases was also greater in the absence of this co-morbidity. As endocarditis is responsible for high morbidity and high mortality, those requiring endocarditis prophylaxis are classified as at-risk patients. Mandatory antibiotic prophylaxis does not completely prevent bacteraemia but does reduce its duration and extent [13] and thus also decreases clinical risks. This is probably the reason for the differences in decision making between patients at risk of endocarditis and healthy patients.

For case vignette 2, almost 70% of the respondents were in favour of therapy. The use of "bone substitute material" was the most favoured therapy (24.8%) and bone block the least favoured (14.9%). Overall, the distribution of the preferred therapy options was much more homogeneous compared to vignette 1. Regardless, a therapy option clearly preferred by most surgeons could not be identified. In addition, the specialist designation exerted no influence on the choice of therapy.

Patient anxiety had hardly any influence on the therapy of choice in case vignette 2. The present bisphosphonate therapy also showed no correlation with the selection of the different therapy options. In this case, bisphosphonate therapy was significantly associated with the general refusal of treatment. Because bisphosphonate therapy has a considerable influence on bone metabolism, it can lead to bisphosphonate-induced bone necrosis [14]. Serious complications are therefore possible under bisphosphonate therapy in the course of implantological treatment [15, 16], and special measures must be taken to minimize risks [17]. These include minimally invasive surgical procedures and antibiotic coverage. This might be the reason for a significantly higher rejection of treatment for patients under bisphosphonate treatment.

One limitation of the study was the low response rate of 46.8%. With the large number of surgeons surveyed, statistical statements could nevertheless be made. The study examined the four descriptors “age”, “need for endocarditis prophylaxis”, “bisphosphonate therapy” and “anxious”. However, there are numerous other reasons that influence surgeons' therapy decisions. Further investigations are therefore necessary.

## Conclusion

In the case of implantological restoration of a single-tooth or multiple-tooth gap, the specialist's designation has little influence on the choice of the preferred therapy. Moreover, patient age and patient fear have virtually no influence on the therapy decision. The therapy options "bone split", "bone block" and "bone replacement material" are selected significantly less frequently for patients requiring endocarditis prophylaxis. Compared to healthy patients, treatment is more frequently refused for patients needing endocarditis prophylaxis or bisphosphonate therapy. The results show that the participants in the survey take into account particular co-morbidities of patients when making therapy decisions.

## Supplementary Information

**Additional file 1: S1 English.** Questionnaire case vignettes.

**Additional file 2: S1 Original.** Questionnaire case vignettes.

**Additional file 3: S2.** Table with raw data.

## Data Availability

The datasets used and/or analysed during the current study are available from the corresponding author on reasonable request.

## Abbreviations

BSM: Bone substitute material
CBCT: Cone beam computed tomography
df: Number of degrees of freedom
Exp(B): Effect coefficient (B)
MFS: Maxillofacial surgeons
mm: Millimetres
N: Number
n.s.: Not significant
OS: Oral surgeons
PRGF: Plasma rich in growth factors
*p* value: Probability
%: Percent
R-squared: Coefficient of determination
S1: Supplemental files
Sig.: Significance
Therapy rec.: Therapy recommended
vs.: Versus
