# Supplementary material for: How do specialist surgeons treat the atrophic tooth gap? A vignette-based study among maxillofacial and oral surgeons
Source: BMC Oral Health. 2021 Jul 3;21:331. doi: 10.1186/s12903-021-01688-9 (PMC8254999; doi:10.1186/s12903-021-01688-9)
Supplement: Supplementary file 1 — Additional file 1: S1 English. Questionnaire case vignettes. [file 12903_2021_1688_MOESM1_ESM.docx]

**Determinants of pre-implantological augmentation procedures – a case vignette study in the severely atrophic single- or multiple-tooth gap**

| **Case 1** | | | |  |
| --- | --- | --- | --- | --- |
| - This is a male patient. Age 52 years/76 years. Tooth 36 is already missing for one year. - **General anamnesis:** No systemic diseases. Currently not in medical treatment/endocarditis prophylaxis required due to artificial heart valves. - **Special anamnesis:** The patient is a non-smoker and is critical about the procedure. - **Clinical findings:** Gap 36 narrowed, gap width 7 mm. - **Radiographic findings (CBCT):** narrow jaw region 36, sufficient bone height - **Referrer’s request:** - The referring dentist wishes to place a single crown on an implant to replace tooth 36.   **CBCT**  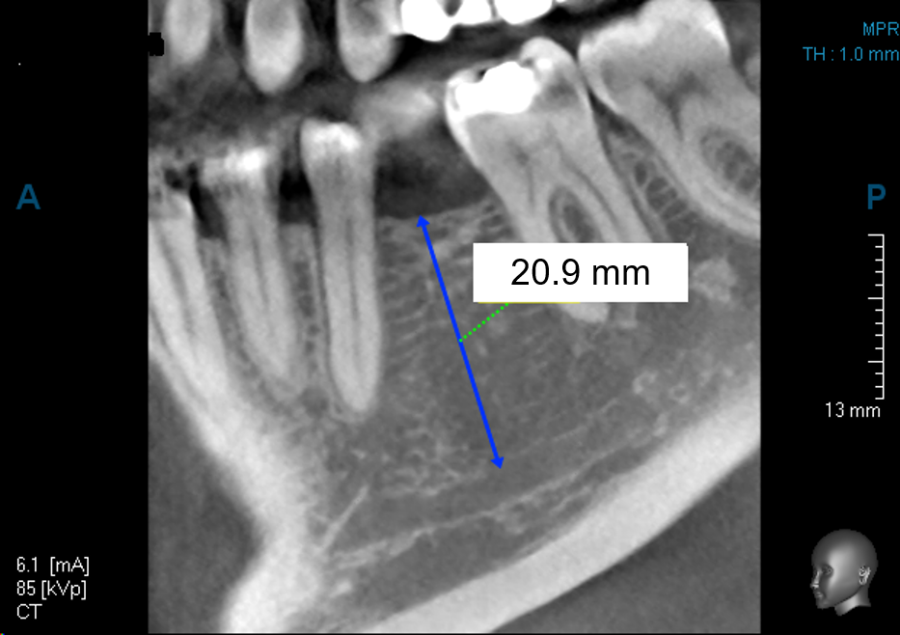  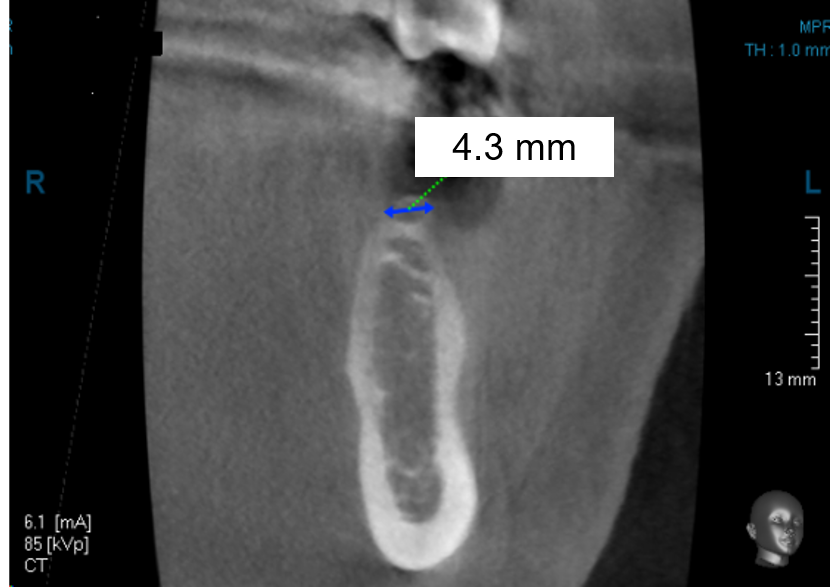 | | | |  |
| **Please analyze the records. How would you proceed surgically in this case?**  **Yes:** This option represents the therapy of my choice (please tick yes only once).  **Not at all:** This therapy is not possible  **Possibly:** I am considering this option. I will decide during surgery. You can select "Possibly" more than once. | **Yes** | **Not at all** | **Possibly** |  |
| Bone Split | 🞏 | 🞏 | 🞏 |  |
| Bone block | 🞏 | 🞏 | 🞏 |  |
| Augmentation with bone substitute material | 🞏 | 🞏 | 🞏 |  |
| Resection | 🞏 | 🞏 | 🞏 |  |
| No therapy | 🞏 | 🞏 | 🞏 |  |
| Other | ------------------------------------------------------------------ | | |  |

| **Case 2** | | | |
| --- | --- | --- | --- |
| - This is a female patient. She is 57 years old. - **General anamnesis:** No systemic diseases. Currently not in medical treatment/FOSAMAX (bisphosphonate) medication due to osteoporosis (1 x weekly 70 mg oral). - **Special anamnesis:** unremarkable - Tooth 35 was not retainable due to a vertical root fracture and was therefore removed. - The patient could not tolerate the temporary denture Regio 35-37. - The patient has a positive attitude towards the procedure / the patient is very anxious. - **Radiographic findings (orthopantomogram and CBCT):** sufficient bone height region 35-37 - **Referrer's request:** The referring dentist would like to place a fixed denture in the lower jaw.   **Orthopantomogram (prior to tooth extraction 35)**  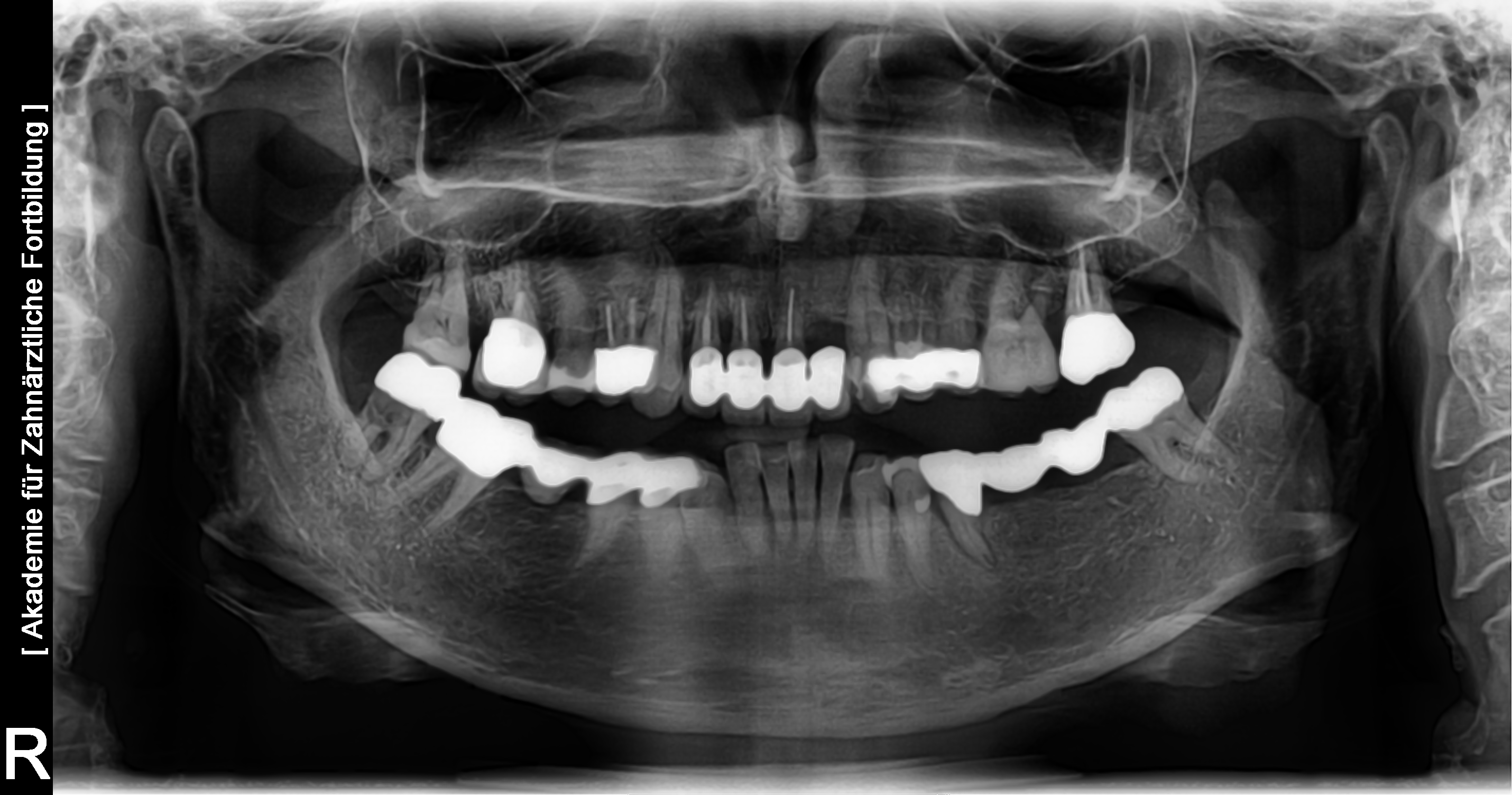 | | | |
| **CBCT (5 months after extraction of tooth 35)**  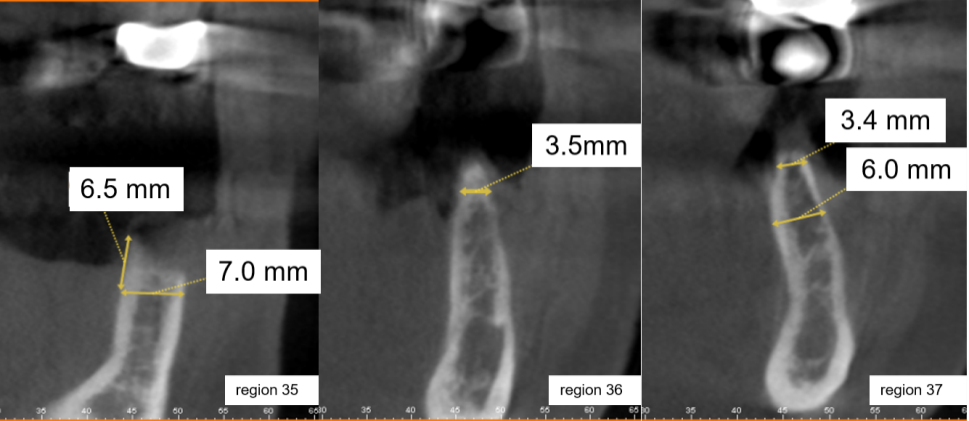  Region 35 Region 36 Region 37 | | | |
| **Please analyze the records. How would you proceed surgically in this case?**  **Yes:** This option represents the therapy of my choice (please tick yes only once).  **Not at all:** This therapy is not possible  **Possibly:** I am considering this option. I will decide during surgery. You can select "Possibly" more than once. | **Yes** | **Not at all** | **Possibly** |
| Bone Split | 🞏 | 🞏 | 🞏 |
| Bone block | 🞏 | 🞏 | 🞏 |
| Augmentation with bone substitute material | 🞏 | 🞏 | 🞏 |
| Resection | 🞏 | 🞏 | 🞏 |
| No therapy | 🞏 | 🞏 | 🞏 |
| Other | ------------------------------------------------------------------ | | |

Commentary to the case vignettes:

The variable descriptors in the anamnesis were combined in this supplementary material. The variables are highlighted in red and green.
