# Supplementary material for: How do specialist surgeons treat the atrophic tooth gap? A vignette-based study among maxillofacial and oral surgeons
Source: BMC Oral Health. 2021 Jul 3;21:331. doi: 10.1186/s12903-021-01688-9 (PMC8254999; doi:10.1186/s12903-021-01688-9)
Supplement: Supplementary file 2 — Additional file 2: S1 Original. Questionnaire case vignettes. [file 12903_2021_1688_MOESM2_ESM.docx]

**Determinanten präimplantologischer Augmentationsverfahren – eine Fallvignettenstudie in der stark atrophischen Einzel- oder Mehrzahnlücke**

| **Fall 1** | | | |  |
| --- | --- | --- | --- | --- |
| - Es handelt sich um einen Patienten. Alter 52 Jahre/76 Jahre. Zahn 36 fehlt seit einem Jahr. - **Allgemeine Anamnese**: Keine systemischen Erkrankungen. Z.Zt. nicht in ärztlicher Behandlung/ Endokarditisprophylaxe erforderlich wegen künstlicher Herzklappen. - **Spezielle Anamnese**: Der Patient ist Nichtraucher und steht dem Eingriff kritisch gegenüber. - **Klinischer Befund**: Lücke 36 eingeengt, Lückenbreite 7 mm - **Röntgenbefund (DVT)**: schmaler Kiefer Regio 36, ausreichende Knochenhöhe - **Anliegen des Überweisers**: Der Überweiser möchte eine Einzelkrone auf einem Implantat zum Ersatz von Zahn 36.   **DVT**  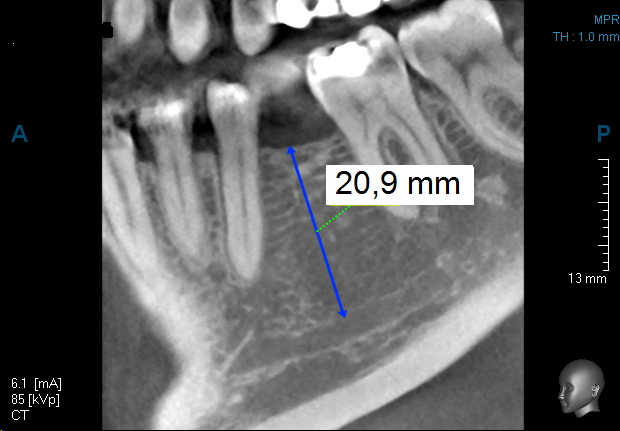  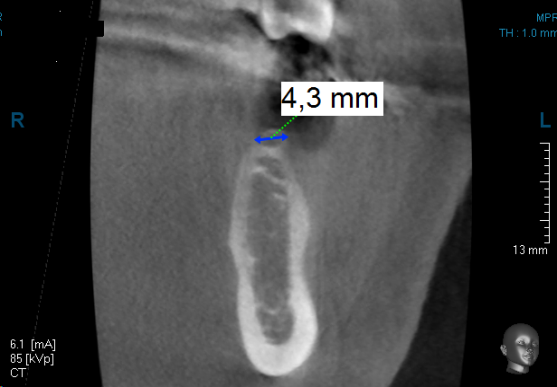 | | | |  |
| **Bitte analysieren Sie die Unterlagen. Wie würden Sie in diesem Fall operativ vorgehen?**  **Ja:** Diese Option stellt die Therapie meiner Wahl dar (bitte nur einmal ja ankreuzen).  **Keinesfalls:** Diese Therapie scheidet aus.  **Möglicherweise:** Diese Option ziehe ich in Erwägung. Ich werde mich intraoperativ entscheiden. „Möglicherweise“ können Sie mehrfach wählen. | **Ja** | **Keinesfalls** | **Möglicherweise** |  |
| Bone Split | 🞏 | 🞏 | 🞏 |  |
| Knochenblock | 🞏 | 🞏 | 🞏 |  |
| Augmentation mit Knochenersatzmaterial | 🞏 | 🞏 | 🞏 |  |
| Resektion | 🞏 | 🞏 | 🞏 |  |
| Keine Therapie | 🞏 | 🞏 | 🞏 |  |
| Sonstiges | ------------------------------------------------------------------ | | |  |

| **Fall 2** | | | |
| --- | --- | --- | --- |
| - Es handelt sich um eine Patientin. Alter 57 Jahre. - **Allgemeine Anamnese**: Keine systemischen Erkrankungen. Z.Zt. nicht in ärztlicher Behandlung/ FOSAMAX-Medikation aufgrund einer vorliegenden Osteoporose (1 x wöchentlich 70 mg oral). - **Spezielle Anamnese:** unauffällig   Der Zahn 35 war aufgrund einer Längsfraktur nicht erhaltungsfähig und wurde entfernt.  Die Patientin kommt mit der Interimsprothese Regio 35-37 nicht zurecht.  Patientin steht dem Eingriff positiv gegenüber/ Patientin ist sehr ängstlich.   - **Röntgenbefund (OPG und DVT):** ausreichende Knochenhöhe Regio 35-37 - **Anliegen des Überweisers**: Der Überweiser möchte einen festsitzenden Zahnersatz im Unterkiefer.   **OPG (vor Extraktion des Zahnes 35)**  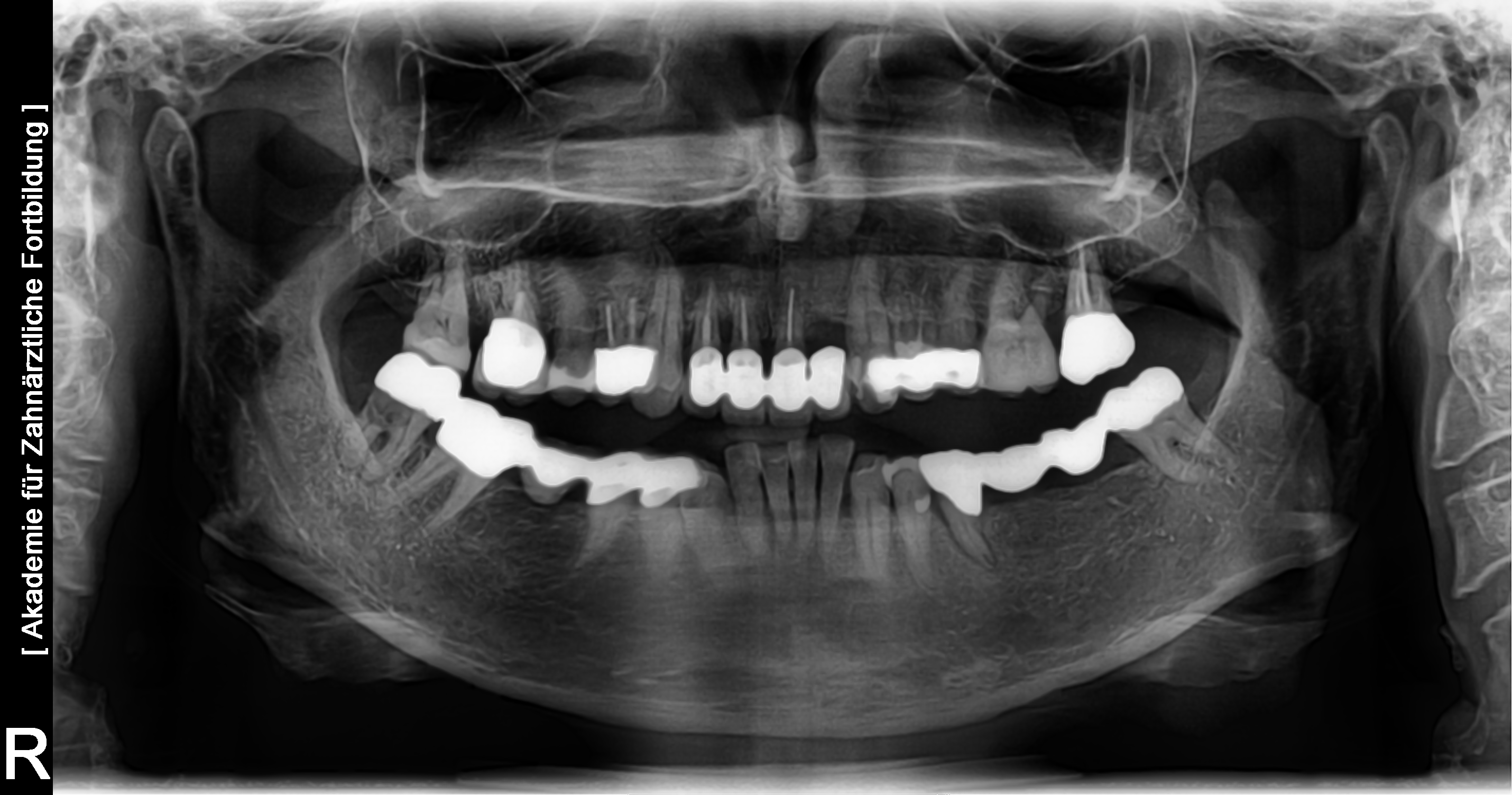  **DVT (5 Monate nach Extraktion des Zahnes 35)**  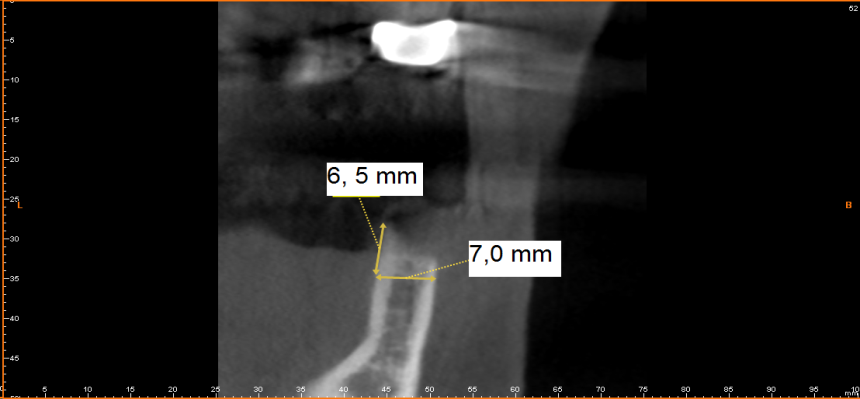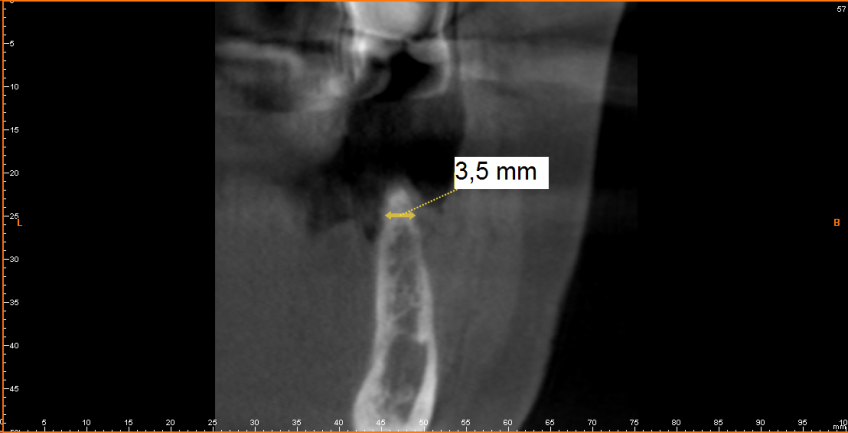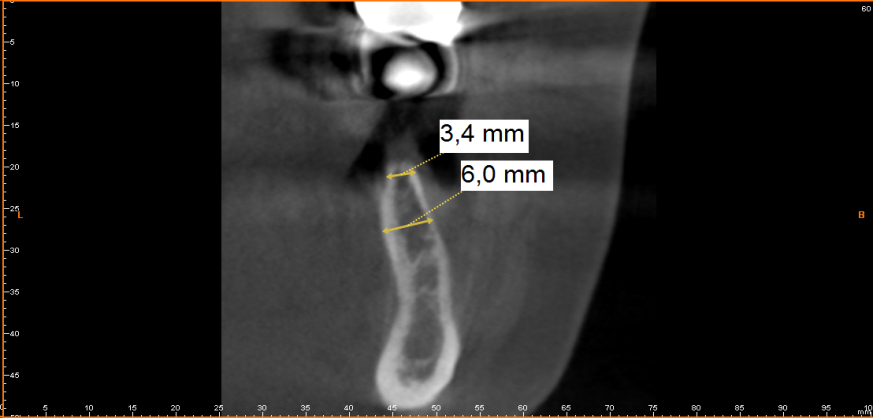  Regio 35 Regio 36 Regio 37 | | | |
| **Bitte analysieren Sie die Unterlagen. Wie würden Sie in diesem Fall operativ vorgehen?**  **Ja:** Diese Option stellt die Therapie meiner Wahl dar (bitte nur einmal ja ankreuzen).  **Keinesfalls:** Diese Therapie scheidet aus.  **Möglicherweise:** Diese Option ziehe ich in Erwägung. Ich werde mich intraoperativ entscheiden. „Möglicherweise“ können Sie mehrfach wählen | **Ja** | **Keinesfalls** | **Möglicherweise** |
| Bone Split | 🞏 | 🞏 | 🞏 |
| Knochenblock | 🞏 | 🞏 | 🞏 |
| Augmentation mit Knochenersatzmaterial | 🞏 | 🞏 | 🞏 |
| Resektion | 🞏 | 🞏 | 🞏 |
| Keine Therapie | 🞏 | 🞏 | 🞏 |
| Sonstiges | ------------------------------------------------------------------ | | |

Kommentar zu den Fallvignetten:

In diesem Ergänzungsmaterial wurden die Variablendeskriptoren der Anamnese zusammengefasst. Die Variablen sind rot und grün markiert.
